# Management of infantile hemangiomas: Recent advances

**DOI:** 10.3389/fonc.2022.1064048

**Published:** 2022-11-29

**Authors:** Wei Xu, Haiguang Zhao

**Affiliations:** Department of Plastic Surgery, Qilu Hospital (Qingdao), Cheeloo College of Medicine, Shandong University, Qingdao, Shandong, China

**Keywords:** infantile hemangioma, diagnosis, treatment, β-blockers, propranolol

## Abstract

Infantile hemangiomas (IHs) are benign vascular tumors commonly observed in children. A small number of cases can manifest as organ or system dysfunction, permanent scarring, or even disfigurement. Currently, diagnosis is mainly based on clinical history, physical examination, and auxiliary inspection. In the treatment of a hemangioma, the functional damage caused by the lesion and complications that may endanger the patient’s life should be given priority. This suggests that identification, diagnosis, and referral to specialists during the early stages of IHs are important factors in preventing related complications and obtaining a better prognosis. During the past few decades, researchers have explored different treatments according to the condition, including oral or topical drugs, topical drug injections, laser surgery, and surgical treatment. However, oral propranolol remains a well-accepted first-line treatment. This article will primarily focus on the recent advances in the clinical diagnosis and treatment of hemangiomas, along with a literature review on the subject.

## 1 Introduction

Infantile hemangiomas (IHs) are common benign vascular and soft-tissue tumors in children (1). The pathological characteristics typically include aberrant endothelial growth and an abnormal blood vessel structure (2). However, the pathogenesis and origin of IHs remain unclear. Many studies have demonstrated that IH formation is a result of a somatic mutation of one or more genes related to endothelial cell proliferation, leading to tumor formation (3). IHs may include single, multiple, segmental, and intermediate types and can also be divided into superficial, deep, and mixed according to the location. In addition, IHs can be complicated by other complications. Therefore, the correct and timely diagnosis of IHs and complications is particularly important in clinical practice.

IHs are usually diagnosed on the basis of medical history and clinical and imaging findings. More than 90% of tumors can be detected by local ultrasound examination. A small number of tumors located in the scalp, sacrocaudal region, and around important organs require magnetic resonance imaging (MRI) examination to understand whether the surrounding tissues and organs are involved and the degree of invasion. Echocardiography can be performed to determine the presence of cardiac insufficiency, cardiac or aortic structural abnormalities in some large or multiple hepatic hemangiomas, and other complications. IHs are mainly treated with systemic and topical drugs supplemented by laser or local injection, which aim to inhibit the proliferation of vascular endothelial cells, promote tumor regression, reduce tumor residue, and prevent functional damage. These therapeutic methods have made great progress; however, in clinical diagnosis and treatment, there are still some unavoidable complications due to systemic administration (4). Multiple studies have established the evidence of IHs; however, we still do not fully understand the pathogenesis and treatment mechanism of IHs. The most urgent requirement for IH treatment is to use the current data to diagnose and classify each child to set up individualized or personalized therapy.

## 2 Epidemiology and risk factors

Generally, IHs often occur in sporadic cases from various regions and racial/ethnic groups, although a few familial aggregations that are considered to be genetically related may exist (5). Recently, a multicenter study found an increased risk of IHs in siblings of patients with IHs, which is considered to be due to the familial clustering of prenatal risk factors (6).

The etiology of IHs is not exactly clear. However, many studies have clarified and confirmed several important risk factors for the development of IHs. They occur more commonly in infants, including low birth-weight babies (7), premature babies, female infants, multiparous pregnancies, estrogen use during pregnancy, a positive family history or genetic evidence, and the Caucasian race. Additional associations included *in vitro* fertilization, maternal smoking, older maternal age, placental anomalies, gestational diabetes, gestational hypertension, vaginal bleeding in the first trimester, amniocentesis, and chorionic villus sampling (8).

## 3 Pathogenesis

The pathogenesis of IHs may be multifactorial, and although scholars have proposed many hypotheses at present, it is still not clearly defined. The pathogenesis of IHs consists mainly of the following:

1. Possible origins of IHs include placental origin and the metastatic niche. Some evidence suggests that many of the characteristic molecular markers of hemangioma vessels are expressed by normal placental fetal microvessels, including merosin, laminin, Lewis Y antigen, Fc gamma receptor II, and erythrocyte-type glucose transporter-1 (GLUT-1) (9). The highly expressed GLUT-1 in the vessels of IHs indicated that IHs were probably derived from placental cells. GLUT-1–positive cells have shown stem cell properties through their ability to differentiate into endothelial cells, pericytes/smooth muscle cells, or adipocytes (10). Notably, IH endothelial cells were negative for aquaporin-1 (AQP1) staining, whereas endothelial cells derived from placental tissue were AQP1 positive (11). Telocytes (TCs), which express AQP1, were regarded as a new tumor cell type of His (12).
2. IHs may occur secondary to perinatal hypoxia. Hypoxic stress may lead to overexpression of angiogenic factors. The important pathway includes hypoxia-inducible factor-1 alpha (HIF-1α), which can increase the transcription of downstream target genes such as GLUT-1 and vascular endothelial growth factor (VEGF) (13, 14). Another pathway closely associated with HIF-1α is the mammalian target of rapamycin, which is closely associated with the protein kinase B pathway. It also upregulates angiogenesis and tumor growth.
3. Vasculogenesis and angiogenesis: hypoxia stimulates IH stem cells to differentiate into endothelial cells to create blood vessels. Tissue ischemia can also stimulate bone marrow-derived endothelial progenitor cells, leading to local vasculogenesis or angiogenesis (15).

Understanding the pathogenesis of IHs lays the foundation for identifying and developing new treatments and for reducing adverse reactions during treatment.

## 4 Clinical characteristics and associated congenital syndromes

IHs are characterized by a predictable course and experience three stages: proliferation, a plateau phase, and involution (16). The morphology of IHs mainly depends on their location. IHs can be classified as deep, superficial, or combined IHs according to their clinical depth. IHs are usually not observed or are often preceded by pale or pink precursor lesions when infants are born. The most rapid proliferation of IHs was completed within 8 weeks after birth. IHs usually grow to their maximum size when the infants are approximately 9–12 months (17); however, the proliferative phase may last longer and take longer to regress in segmental or deep IHs. After a short plateau phase, IHs gradually regress after 1 year of age, and most may be in complete involution when the child is 4 years old (18). In this phase, some cases present with skin problems such as significant permanent scarring, dilation of the capillaries, and other changes.

Although IHs are usually small and harmless, in a significant proportion of patients, they can lead to serious complications during the rapid proliferation phase, including pain, ulceration, functional impairment, cosmetic disfigurement, and potentially serious visceral abnormalities. Furthermore, a few cases of IHs can be associated with additional abnormalities. PHACE(S) and LUMBAR are considered the two most recognized syndromes.

PHACE(S) is characterized by posterior fossa vascular malformation, segmental hemangiomas of the face, arterial anomalies, coarctation of the aorta, cardiac anomalies, eye abnormalities, and, insome cases, sternal defects or supraumbilical raphe defects. The LUMBAR syndrome is usually characterized by lower-body hemangioma, urogenital abnormalities, ulceration, myelopathy, bony deformities, and anorectal malformations (19).

## 5 Diagnosis

IHs are diagnosed clinically; the evaluation of infants with IHs should include a detailed history and a full-body physical examination, and large or potentially problematic lesions should be confirmed by a specialist after presenting a systematic analysis. The rapidly proliferating nature of IHs can lead to misdiagnosis in patients with malignancy, as they may mimic dermatofibrosarcoma and infantile fibrosarcoma (15). Nevus simplex and port wine stains are flat, hyperpigmented capillary malformations, typically on the head and neck regions of newborns, that may resemble IHs. If the lesion was present at birth and has not grown, it may be a congenital hemangioma rather than IHs. Other benign vascular tumors include spindle-cell, epithelioid, intramuscular, and pyogenic granulomas (20).

IHs in a beard distribution/location should prompt an evaluation of the airway, despite IHs rarely causing airway blockage. Five or more cutaneous hemangiomas are associated with the presence of IHs involving the liver, and ultrasound can be helpful in diagnosis. Moreover, large or multiple IHs in the liver increase the risk of high-output cardiac failure, as well as acquired hypothyroidism (21).

PHACE(S) syndrome is not always large, and smaller segmental IHs on the face should increase the possibility of associated anomalies (22). Many studies have suggested that head and neck MRI with magnetic resonance angiography should be performed in infants with segmental facial hemangiomas of ≥5 cm. In addition to MRI/magnetic resonance angiography, patients with PHACE(S) syndrome should undergo echocardiography and ophthalmologic assessment. Patients with LUMBAR syndrome also need to undergo an MRI of the pelvic and lower spine (23, 24).

## 6 Treatment

Treatment should be considered comprehensively after the diagnosis of IHs. Most IHs do not require active treatment because of their natural course but do require regular follow-up. However, high-risk IHs are identified according to their size, nature, or location; therefore, active interventions are needed. Urgent referral and early intervention are recommended for infants with high-risk IHs, including lesions associated with life-threatening complications, functional impairment, ulceration or bleeding, structural abnormalities (PHACE or LUMBAR syndrome), and permanent disfigurement (25).

Various systemic and topical therapies have been investigated for IHs treatment. The current systemic therapy for IHs can safely start with propranolol. Other systemic treatments for IHs have been reported to include corticosteroids and other β-blockers (26).

### 6.1 Systemic treatments

#### 6.1.1 Propranolol

Propranolol is recognized as a first-line drug for IH treatment. The mechanism of propranolol in the treatment of IHs is unclear. Generally, it is believed that there are three stages of drug efficacies: (1) the early effect is to reduce the release of nitric oxide and cause vasoconstriction (within 1 to 3 days after the start of treatment); (2) in the middle stage, it inhibits angiogenesis by blocking pro-angiogenic signals; and (3) in the late stage, it induces endothelial cell apoptosis to obtain a long-term therapeutic effect (27). AQP1 has been identified as a major driver of the antitumor response to propranolol by decreasing capillary-like tube formation, inducing apoptosis, inhibiting nitric oxide production, and regulating the renin-angiotensin system (12). In 2008, two infants with IHs were administered propranolol to treat cardiac indications, which showed beneficial effects; beta-blockers gradually replaced glucocorticosteroids and became the first line of treatment. Compared with previous treatments, the efficacy of propranolol in the treatment of IHs is clear, with a well-tolerated and effective treatment, especially in the proliferative phase (28, 29).

Although propranolol is effective in most cases, rebound growth after discontinuation is relatively common and a small number of IHs are resistant to propranolol (30, 31). Propranolol-resistant IHs were defined as failure to achieve the expected therapeutic response to propranolol after oral administration of propranolol ≥ 2 mg/kg/day for at least 4 weeks, that is, sustained growth during the proliferative phase or no shrinkage during the postproliferative phase. The incidence of propranolol resistance has been estimated to be 1%. Moreover, recent studies have suggested that propranolol still causes side effects (32, 33), of which sleep disturbances and agitation are the most common. Other potentially serious side effects include hypoglycemia, hypotension, bradycardia, bronchospasm, peripheral vasoconstriction, and diarrhea. Additionally, because propranolol has only been used for a dozen years since its discovery, its potential long-term effects may not be apparent. However, Thai et al. suggested that propranolol increased the central nervous system effects and the risks associated with sleep (34). In addition, new dosage forms to reduce the adverse effects of systemic and topical propranolol therapy and to increase therapeutic efficacy are also under investigation (35, 36).

#### 6.1.2 Other beta-blockers

Studies have revealed that nadolol can be an alternative option because of its high efficiency and safety when propranolol therapy is ineffective, shows adverse effects, or requires faster outcomes (37). However, McGillis elaborated on a death associated with the use of nadolol for IHs, as the drug was left in the gastrointestinal tract and not excreted in time, causing continuous drug absorption (38). Therefore, it is necessary to improve the safety of nadolol in the treatment of IHs and to guide parents to pay more attention to the stools of children. Intervention and treatment should be performed as soon as possible when symptoms occur.

In recent years, oral atenolol has been suggested to have effects similar to those of oral propranolol (39). A recent large randomized clinical trial revealed that atenolol has similar efficacy and fewer adverse events in the treatment of infants with problematic IHs when compared with propranolol. Oral atenolol can be used as an alternative treatment option for IH patients requiring systemic therapy (40). Owing to its long half-life, the frequency of administration can be reduced, which also displays a broad and promising application prospect (41). As atenolol is a selective β-2 blocker, it may reduce the risk of asthma, hypoglycemia, sleep disturbances, and cognitive effects. Notably, atenolol also seems to have a faster therapeutic effect on ulcerative IHs (42–45).

#### 6.1.3 Corticosteroids

Before propranolol was used as a first-line treatment because of its better outcomes and fewer side effects, systemic corticosteroids were the mainstay of treatment for IHs (46). Systemic corticosteroids may be prescribed to treat patients in whom beta-blocker therapy is contraindicated or there is an inadequate response to oral propranolol.

According to Aly et al., compared to monotherapy with corticosteroids, a combination with propranolol may expedite the involution of IHs and exert a better treatment effect, especially for IHs requiring urgent treatment (47). Notably, Gnarra proposed that low doses of propranolol combined with prednisone in segmental IHs in the PHACES syndrome can negate each other, thereby reducing the side effects of monotherapy (48).

#### 6.1.4 Other drugs

Other systemic treatment options include captopril, intralesional pingyangmycin, and intravenous vincristine; however, owing to the incidence of adverse reactions and side effects in infants, the frequency of application is not high.

### 6.2 Topical therapy

Topical timolol is used to manage small, superficial IHs. Timolol maleate 0.5% represents a well-tolerated, safe, and efficacious treatment and is seemingly more effective in the proliferative phase of IHs than in the mature phase when applied to small and superficial IHs (39, 49). Topical therapy is considered an adjunctive treatment during the observation period of low-risk IHs.

### 6.3 Laser therapy

Laser therapy is a well-tolerated local treatment that is often used as an alternative treatment for IHs (50). Pulsed dye laser (PDL) or long-pulse Nd : YAG, which are based on the selective photothermolysis theory, are considered the most common laser therapies for IHs (51). The main side effect is that some lesions may have blisters after laser irradiation, which requires parents to apply ice in time and avoid sun exposure in cases of pigmentation (52). According to the current literature (53), PDL, with a penetration depth of approximately 1.5 mm, is mainly used for superficial IHs or in combination with Nd : YAG and other treatments for other IHs. Nd : YAG can reach a penetration depth of 10 mm and is mainly used in infants with propranolol intolerance. The combination of laser therapy with other treatments provides significantly better outcomes than a single treatment. Multiple laser therapies combined with systemic propranolol therapy may have advantages over monotherapy (54). A meta-analysis also demonstrated that topical timolol combined with laser therapy is more effective and causes fewer adverse reactions than monotherapy (55).

### 6.4 Other treatments

Surgical intervention has also been used commonly in the past and is now being considered for IHs that are more dangerous or dysfunction, such as airway obstruction and heart failure. Direct drug delivery into the tumor is another popular treatment for vascular diseases, and it is frequently combined with other treatments to obtain better effects. However, sclerotherapy is rarely used to treat facial IHs, which may cause ulceration and scar formation. Combination sclerotherapy with PDL and Nd : YAG laser treatment is demonstrably effective, which reduces adverse reactions and shortens the treatment time. Different types of IHs can be treated according to the tumor depth with different depths of laser penetration (56). It has been reported that lauromacrogol combined with triamcinolone intralesional injection of IHs also achieves good therapeutic effects (57).

## 7 Conclusion

With a better understanding of the natural course of IHs and related complications, the efficacy of propranolol and its mechanism of action are clearer, which helps enhance further research on them as well as the treatment of IHs. In many cases, treatment decisions are based on the presence or absence of complications. Infants who may develop complications and other risks should be considered and treated by a specialist clinic as soon as possible. After a comprehensive evaluation by a specialist, appropriate and individualized approaches should be used to treat or prevent complications. Propranolol is the mainstay of treatment, and combination treatment has unique advantages. Promising results have been reported during the exploration of new treatment options.

## Author contributions

XW: conceptualization, methodology, data curation, and writing—original draft preparation. HZ: validation and writing—review and editing. All authors have read and agreed to the published version of the manuscript.

## Funding

This work was supported by the Shandong Medical Trade Union Committee (SDYWZGKCJH2022002) and the Qingdao Municipal Health Commission (2022-zyym42).

## Conflict of interest

The authors declare that the research was conducted in the absence of any commercial or financial relationships that could be construed as a potential conflict of interest.

## Publisher’s note

All claims expressed in this article are solely those of the authors and do not necessarily represent those of their affiliated organizations, or those of the publisher, the editors and the reviewers. Any product that may be evaluated in this article, or claim that may be made by its manufacturer, is not guaranteed or endorsed by the publisher.
